# Stimulated Organic Carbon Cycling and Microbial Community Shift Driven by a Simulated Cold-Seep Eruption

**DOI:** 10.1128/mbio.00087-22

**Published:** 2022-03-01

**Authors:** Yongxin Lv, Shanshan Yang, Xiang Xiao, Yu Zhang

**Affiliations:** a School of Oceanography, Shanghai Jiao Tong University, Shanghai, People’s Republic of China; b State Key of Laboratory of Ocean Engineering, Shanghai Jiao Tong University, Shanghai, People’s Republic of China; c School of Life Sciences and Biotechnology, Shanghai Jiao Tong University, Shanghai, People’s Republic of China; d International Center for Deep Life Investigation (IC-DLI), Shanghai Jiao Tong University, Shanghai, People’s Republic of China; e Laboratory for Marine Biology and Biotechnology, Pilot National Laboratory for Marine Science and Technology, Qingdao, People’s Republic of China; f College of Marine Science and Technology, China University of Geosciences, Wuhan, People’s Republic of China; g Shenzhen Yuchi Inspection & Testing Technology Co., Ltd., Shenzhen, People’s Republic of China; Corporación CorpoGen

**Keywords:** cold-seep microbiome, methane oxidation, methane partial pressure

## Abstract

Cold seeps are a major methane source in marine systems, and microbe-mediated anaerobic oxidation of methane (AOM) serves as an effective barrier for preventing methane emissions from sediment to water. However, how the periodic eruption of cold seeps drives the microbial community shift and further affects carbon cycling has been largely neglected, mainly due to the technical challenge of analyzing the *in situ* communities undergoing such geological events. Using a continuously running high-pressure bioreactor to simulate these events, we found that under the condition of simulated eruptions, the abundance of AOM-related species decreased, and some methane was oxidized to methyl compounds to feed heterotrophs. The methanogenic archaeon *Methanolobus* replaced ANME-2a as the dominant archaeal group; moreover, the levels of methylotrophic bacteria, such as Pseudomonas, *Halomonas*, and *Methylobacter*, quickly increased, while those of sulfate-reducing bacteria decreased. According to the genomic analysis, *Methylobacter* played an important role in incomplete methane oxidation during eruptions; this process was catalyzed by the genes *pmoABC* under anaerobic conditions when the methane pressure was high, possibly generating organic carbon. Additionally, the findings showed that methyl compounds can also be released to the environment during methanogenesis and AOM under eruption conditions when the methane pressure is high.

## INTRODUCTION

Cold seeps are the areas of seepage of hydrogen sulfide, methane, and other hydrocarbon-rich fluids, which originate from tens of meters to kilometers below the seafloor (1, 2). The ecosystem here mainly depends on the anaerobic oxidation of methane (AOM) acting as an energy source, together with the organic carbon input from the water column (3). *In vitro* studies suggest that anaerobic methanotrophic archaea (ANME) exhibit AOM activity and are mostly associated with sulfate-reducing bacteria (SRB), which oxidize methane to bicarbonate or acetate for utilization by heterotrophs (4–7). Moreover, AOM is an important barrier preventing methane emissions from anoxic sediments to water columns. It is estimated that up to 300 Tg of methane per year, ∼88% of the methane from the subsurface, is consumed through AOM (8). This number may be underestimated since recent research has demonstrated that 3 to ∼25% of methane consumed by ANME goes to acetate and thus contributes to the organic carbon pool (5). Considering that the AOM process is the only pathway to convert methane into carbon compounds that can be utilized by life in anoxic zones, the methane budget and the carbon bioavailability in anoxic cold seep sediment are directly affected by AOM (9, 10). In addition, the global distribution along active and passive margins and substance exchange among the seafloor make cold seeps important for the biogeochemical cycle (1, 11, 12).

However, most of the research has focused on stable cold seeps. Cold seeps along the active margins would exhibit many geologic activities, and the resulting eruptions would ultimately affect the ecosystem and disturb AOM activity (12). A series of studies on the Håkon Mosby Mud Volcano showed that eruptions would increase gas and fluid emissions and significantly impact faunal density, microbial activity, and environmental variables (13–15). The sediment was observed to have rapid uplift and fall caused by the expansion and release of trapped gases (15). There was a similar phenomenon in “HaiMa” cold seeps, with a larger movement scale (16). In addition, organism distribution, formation of gas hydrate, precipitation of carbonate, and generation of new fractures are also affected by gas emissions, and eruption influences gas emissions (16). In summary, the seafloor morphology, chemosynthetic taxa in cold seeps, and metabolic activities are significantly affected by natural disturbances (17). However, due to technical challenges in directly monitoring dramatically unstable environments, limited investigations have been carried out. The exact community shifts and changes in AOM activity during the eruption remain unrevealed.

In this research, we took an alternative approach using a continuously running high-pressure bioreactor to simulate cold-seep eruption. As previously described, this reactor is specifically designed to simulate the erupting environments in the deep sea, such as cold seeps and hydrothermal vents (18). It has been successfully used to sustain and to investigate the ANME/SRB community (19). Basically, in this system, we control the methane partial pressure and incubation pressure independently to simulate the gas emission and vertical lift caused by eruption. The AOM activity and the microbial community were closely monitored during the experiment. Here, we systematically illustrate the ecological impacts on AOM activity, microbial community structure, and microbial metabolism driven by cold-seep eruptions.

## RESULTS

### Dynamic changes in AOM activity along the simulated eruption.

In this study, we simulated a cold-seep eruption event to investigate its effect on the microbial community. The original sample was collected from a mud volcano in the Gulf of Cadiz that is rich in methane and contributes to the cold-seep ecosystem (19–21). The sample was first cultivated for 8 years under a condition in which methane and sulfate were supplied as the only energy sources to enrich AOM-related species. The sample was then incubated in a continuously running high-pressure bioreactor that could independently control the methane partial pressure (MPP) and incubation pressure (IP), and the highly active AOM community was enriched and used as the biomass for the following experiments (18). During the incubation, continuous flow removed the metabolic products to avoid their inhibition of AOM activity. At the same time, it prevented the toxicity of sulfide accumulation on SRB (22). Six incubations were carried out, and each lasted for 2 months. The incubation conditions are shown in Table 1 and Materials and Methods in detail. Briefly, there were two stages with different MPPs. In the first stage, which contained the first four incubations, the MPP was set to 8 MPa to serve as a control and identify the effect of the incubation pressure independently. The L8II incubation was set for the recovery of the microbial community, which suffered from high incubation pressure during the two previous incubations. The MPP was then elevated to 12 MPa in the second stage to simulate cold-seep eruption events. During each stage, the IP was adjusted to mimic sediment movement.

**TABLE 1:** Experimental design showing the incubation conditions

Stage	Incubation name	IP (MPa)	MPP (MPa)	Time (mo)
Pre-I	Origin			96
I	L8	8	8	2
	L15	15	8	2
	L30	30	8	2
	L8II	8	8	2
II	H15	15	12	2
	H30	30	12	2

**FIG 1:**
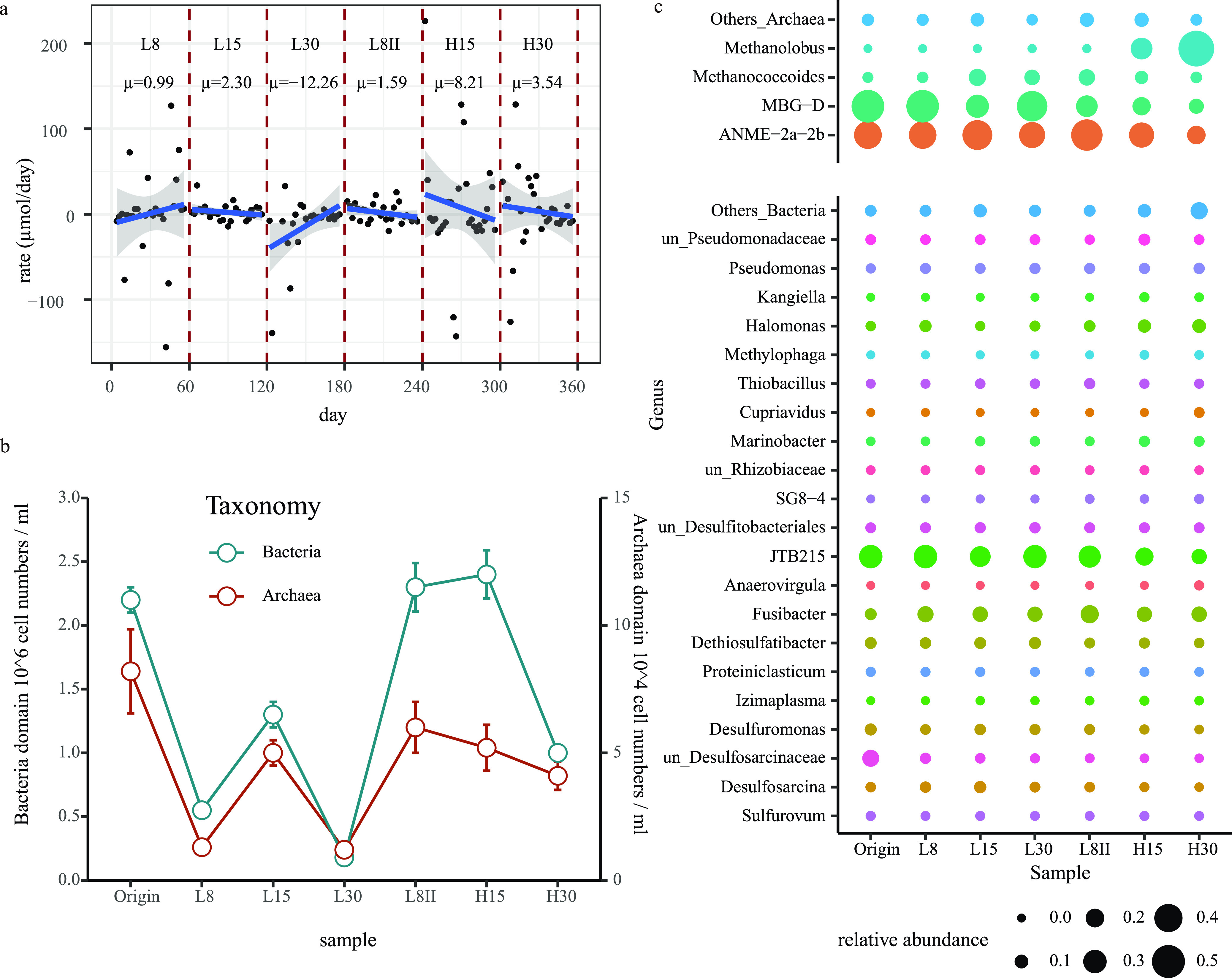
Microbial activities and cellular quantifications under different incubation conditions. (a) The mean value (μ) and standard deviation of sulfide production rate were calculated based on *n* values of 27 (L8), 28 (L15), 25 (L30), 28 (L8II), 28 (H15), and 28 (H30). (b) The mean value and standard deviation of archaeal and bacterial cell numbers were calculated based on an *n* value of 3. (c) Microbial community structure shift during incubation. The point size shows the relative abundance of each genus for bacteria (bottom) and archaea (top). Different colors represent the different genera, and genera whose relative abundances were <0.01 were grouped into “Others.”

To monitor the AOM activity, the key compound concentrations were measured every 2 days, and the results showed that the AOM activity was controlled by both IP and MPP (Fig. 1a; see also Table S1 in the supplemental material). As indicated by the production of sulfide, the optimal IP was 15 MPa, which is in accordance with the original water depth (1,200 m) of the inoculum. The increased MPP, simulating the eruption, may promote this and had apparent benefit for the community to cope with high IP. For example, under 15 MPa of IP, sulfide production increased from 2.30 to 8.21 μmol per day when the MPP was increased from 8 to 12 MPa (Fig. 1a). There was no sulfide production under 30 MPa of IP (L30) when the MPP was low, but apparent activity was detected when the MPP was adjusted to 12 MPa (H30). A larger biomass observed between the same two incubations may also suggest a stronger resistance against a high IP (Fig. 1b and Table S2).

10.1128/mbio.00087-22.3 TABLE S1 Reaction rate and key compound concentrations. Download Table S1, XLSX file, 0.02 MB. Copyright © 2022 Lv et al. 2022 Lv et al. https://creativecommons.org/licenses/by/4.0/ This content is distributed under the terms of the Creative Commons Attribution 4.0 International license.

10.1128/mbio.00087-22.4 TABLE S2 Cell numbers of key genera in each incubation, calculated by archaeal/bacterial biomass times relative to the amplicon abundance based on sequencing. Download Table S2, XLSX file, 0.02 MB. Copyright © 2022 Lv et al. 2022 Lv et al. https://creativecommons.org/licenses/by/4.0/ This content is distributed under the terms of the Creative Commons Attribution 4.0 International license.

### In-depth investigation of the community composition.

**FIG 2:**
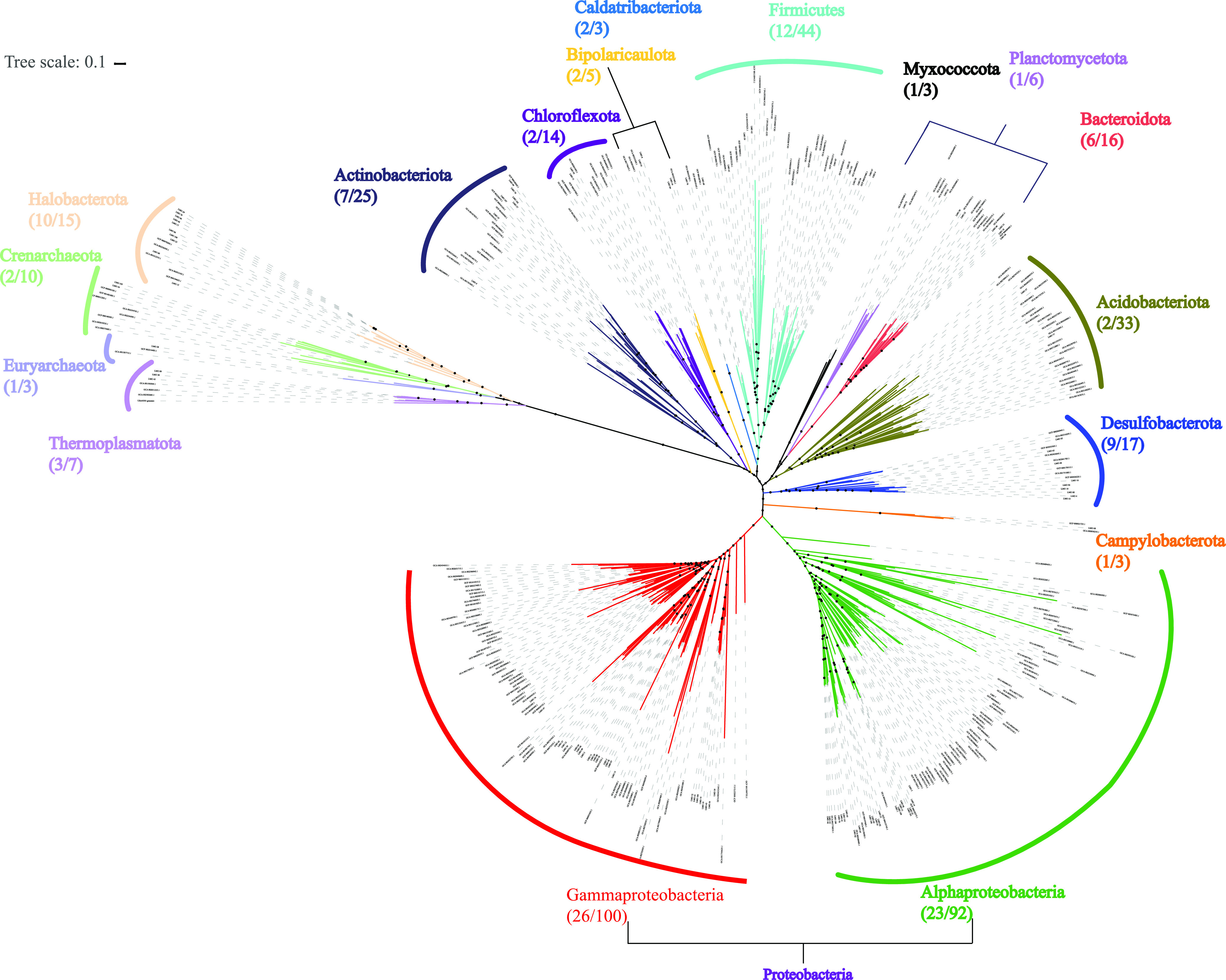
Phylogenetic tree of retrieved metagenome-assembled genomes (MAGs) based on concatenated conserved proteins. A total of 40 conserved genes were used to generate a maximum likelihood tree. Bootstrap support is indicated by the size of the black dots, and those representing 70% are depicted. The numbers under the phylum names represent the numbers of retrieved MAGs in this study (left) and reference genomes (right).

Both metagenome and amplicon sequencing were performed at the end of the incubation and with the original cultivated sample (Origin). For amplicon data sets, the SILVA 138 database (23) and QIIME2 (24) were used to determine the taxonomic composition and diversity. For metagenome data sets, a genome-centric analysis was also performed to illustrate the community structure at the taxonomic level. A total of 1,766,410,042 clean reads remained after quality control and were used in the coassembly. A total of 170,472 scaffolds were obtained after sequences of <500 bp were filtered. More than 99% of reads could be successfully mapped to these scaffolds and resulted in a range of average sequencing depths from 50.32 to 71.79 (Table S3). A total of 110 metagenome-assembled genomes (MAGs) were reconstructed with 80 high-quality drafts (completeness > 90%) and 28 medium-quality drafts (completeness between 50% and 89.9%) based on the MixS standard (25), which covers 19 phyla and nearly 80% of all reads (Fig. 2 and Table S3). ANME-2a and *Methanolobus* are the dominant archaeal groups, while *Desulfobacterota* and *Gammaproteobacteria* are the dominant bacterial members.

10.1128/mbio.00087-22.5 TABLE S3 Statistics of the metagenomic assembly and each metagenome-assembled genome. Download Table S3, XLSX file, 0.08 MB. Copyright © 2022 Lv et al. 2022 Lv et al. https://creativecommons.org/licenses/by/4.0/ This content is distributed under the terms of the Creative Commons Attribution 4.0 International license.

Moreover, 30 out of 80 high-quality drafts were identified as novel taxa with no reference genome in the Genome Taxonomy Database (GTDB) database (26). In addition to the ANME-2a MAG reported in previous research (5) (Cadiz_LYX17), another two ANME-2a MAGs were identified in this study (Cadiz_LYX29 and Cadiz_LYX78).

### Effect of IP on the microbial activity and community.

Using the continuous bioreactor, we were able to reveal the effects of the IP and MPP independently. In general, the microbial sensitivity to IP defines the water depth of their natural habitat, and the MPP defines the Gibbs free energy in the AOM reaction, which could be utilized for microbial growth. In this study, we set the IP to 8, 15, and 30 MPa to mimic the vertical migration of the active AOM community driven by the eruption. The community had the highest biomass with an IP of 15 MPa, which is similar to the pressure at its original sampling site (1,200-m water depth), while increases in the IP to 30 MPa caused apparent decreases in biomass and AOM activity under both low and high MPP (Fig. 1a and b).

### Effect of MPP on the microbial activity and community.

**FIG 3:**
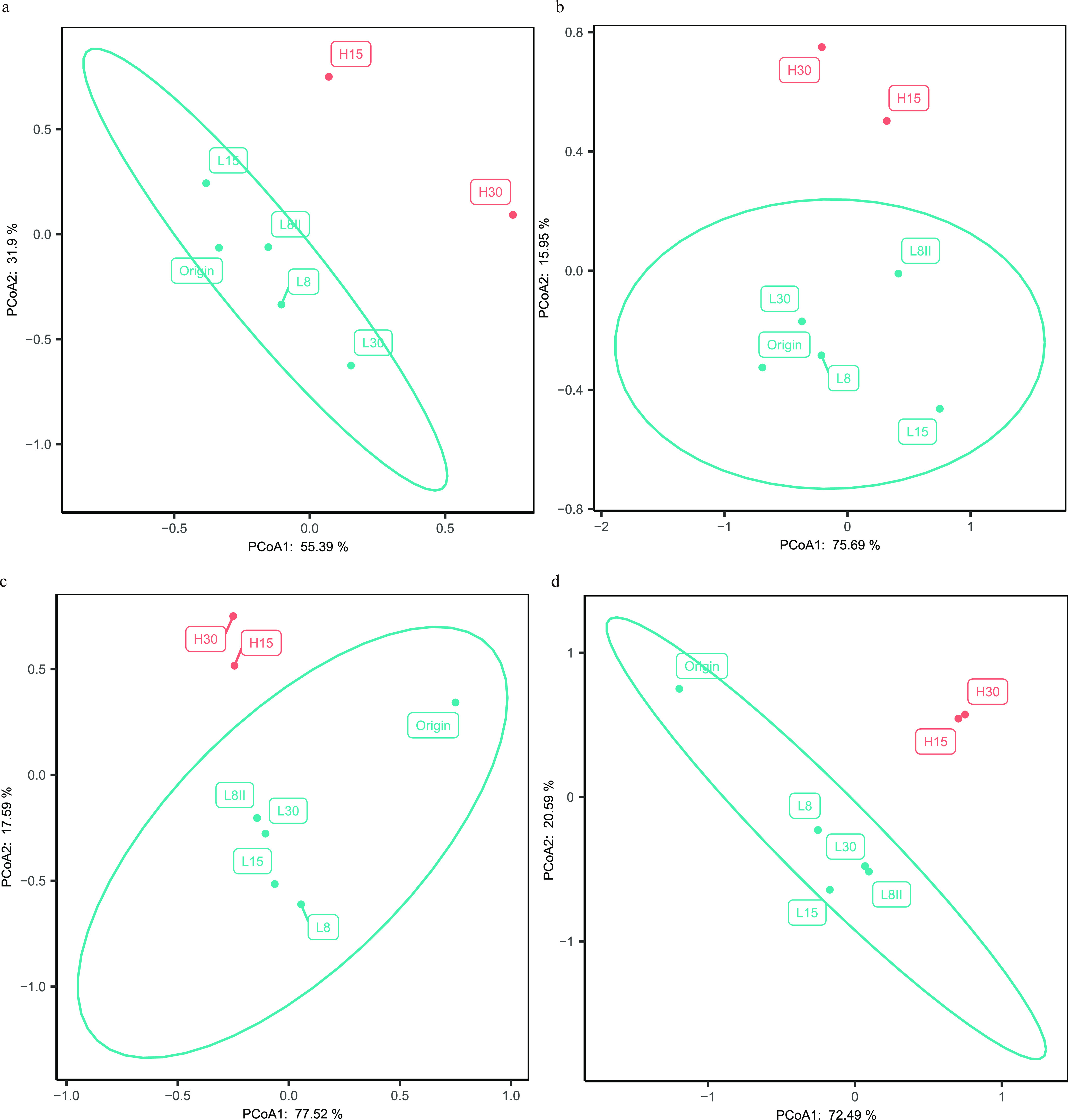
Principal-component analysis (PCoA) of incubations with different conditions, as follows: Based on the read number of each archaeal amplicon sequence variant (ASV) (a), based on the read number of each bacterial ASV (b), based on the relative abundances of 43 abundant MAGs (c), and based on the relative abundances of KEGG orthologs (d).

MPP mostly influenced the taxonomic composition and further metabolic functions (Fig. 3). Most previously dominant species were strongly stirred under higher MPP, especially ANME-2a-2b and SRB (Fig. S1). *Methanolobus* replaced ANME-2a-2b as the dominant archaeal group under higher MPP (Fig. 1c). Bacterial groups that reduce inorganic sulfur compounds, including *Desulfobacteraceae*, *Defulfitobacteriales*, and *Desulfuromonas*, were dominant but continued to decrease over the experimental period, especially when the MPP was changed to 12 MPa. A similar trend was observed with *Clostridia* JTB215. The abundance of another abundant group, *Fusibacter*, increased when the IP was adjusted to 8 MPa both the first time (from 6.37% to 14.98%) and the second time (from 13.03% to 19.21%). In addition, the abundance of Pseudomonas was relatively stable (6.67% to ∼11.08%). In addition, methylotrophic MAGs increased under higher MPP, such as Pseudomonas (Cadiz_LYX64 and Cadiz_LYX94), *Halomonas* (Cadiz_LYX97 and Cadiz_LYX58), and *Methylobacter* (Cadiz_LYX74) (27, 28), while *Firmicutes* (mainly *Clostridia*) decreased, suggesting a distinct primary energy process and the emergence of a heterotrophic community.

10.1128/mbio.00087-22.1 FIG S1 Biomass under different incubation conditions. Download FIG S1, PDF file, 0.01 MB. Copyright © 2022 Lv et al. 2022 Lv et al. https://creativecommons.org/licenses/by/4.0/ This content is distributed under the terms of the Creative Commons Attribution 4.0 International license.

**FIG 4:**
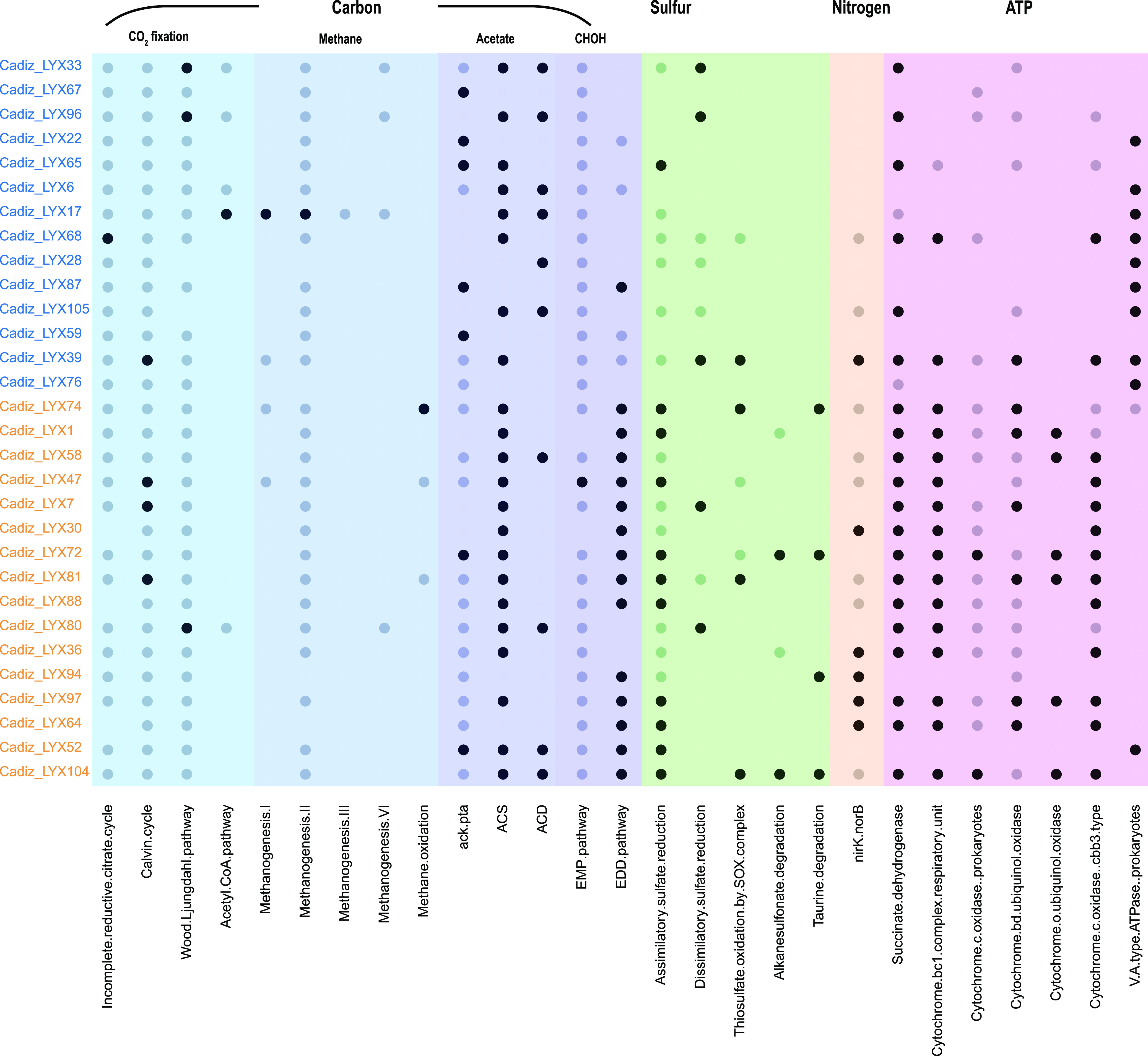
Metabolic potential of 30 abundant MAGs. A black dot indicates a complete or nearly complete pathway (>0.75), a gray dot only partial gene existence (≥0.2 and <0.75), and a blank a lack of related genes (<0.2). MAGs with blue color would have a lower relative abundance in incubation H15, and MAGs with orange color would have a larger relative abundance. EMP, Embden-Meyerhof-Parnas; EDD, Entner-Doudoroff.

To verify this finding, an analysis of the key genes and metabolic modules was performed. A custom Python script, which is similar to KEGG-Decoder (29) but expanded to more modules and functions, was used to evaluate the related metabolic ability of 30 abundant MAGs (whose difference in abundance between incubation L15 and H15 was >0.1%) (Fig. 4 and Table S3). MAG “Cadiz_LYX17,” which belonged to ANME-2a and was the only abundant archaeal participant in AOM, decreased under higher MPP. This was consistent with the amplicon sequencing results. Similarly, the relative abundance of 4 out of 6 MAGs that had a complete dissimilatory sulfate reduction (DSR) pathway decreased. However, one of them belonged to *Thiobacillus* (Cadiz_LYX39), which reportedly carries out the reverse DSR (30). At the same time, although MAG Cadiz_LYX65 also belonged to *Desulfobacterota* and was mostly complete (99.32%), none of the DSR-related genes were identified. It was found that the system became more diversified in terms of metabolic potential, especially carbon flow, under the simulated erupting conditions. Larger abundances of the formaldehyde assimilation pathway (Entner-Doudoroff [EDD] pathway) and methylotrophs indicate that there would be methyl-like substances for the emerging heterotrophs to utilize (Fig. 4 and Fig. S2).

10.1128/mbio.00087-22.2 FIG S2 Abundance of key metabolic genes between incubations from nonerupting conditions and erupting conditions. Download FIG S2, PDF file, 0.4 MB. Copyright © 2022 Lv et al. 2022 Lv et al. https://creativecommons.org/licenses/by/4.0/ This content is distributed under the terms of the Creative Commons Attribution 4.0 International license.

The metabolic pathways associated with sulfur in the community also became more complex. Genes related to assimilatory sulfate reduction and thiosulfate oxidation, which mainly belonged to Pseudomonas_A (Cadiz_LYX64 and Cadiz_LYX94), *Cupriavidus* (Cadiz_LYX104), and *Marinobacter* (Cadiz_LYX88 and Cadiz_30), were more enriched under erupting conditions. In addition, *Cupriavidus* and *Ralstonia* (Cadiz_LYX72) also degrade alkanesulfonate and taurine to sulfite, which is then reduced to sulfide and cysteine (Fig. 4). In addition, a higher abundance of transporter genes for these three sulfur compounds also showed enhanced assimilation (Fig. S2). Combined with the enhanced assimilation, higher energy input, and biomass, the community would have a better resistance to a higher IP under erupting conditions (H30) than under nonerupting conditions (L30). Higher Shannon and Pielou diversity indices were also observed (Table S4), which could aid the stability of microbial communities in the face of fluctuations in environmental factors (31).

10.1128/mbio.00087-22.6 TABLE S4 Diversity and relative abundances of archaeal groups, bacterial groups, and metagenome-assembled genomes. Download Table S4, XLSX file, 0.06 MB. Copyright © 2022 Lv et al. 2022 Lv et al. https://creativecommons.org/licenses/by/4.0/ This content is distributed under the terms of the Creative Commons Attribution 4.0 International license.

### Conceptual model of the community shift.

**FIG 5:**
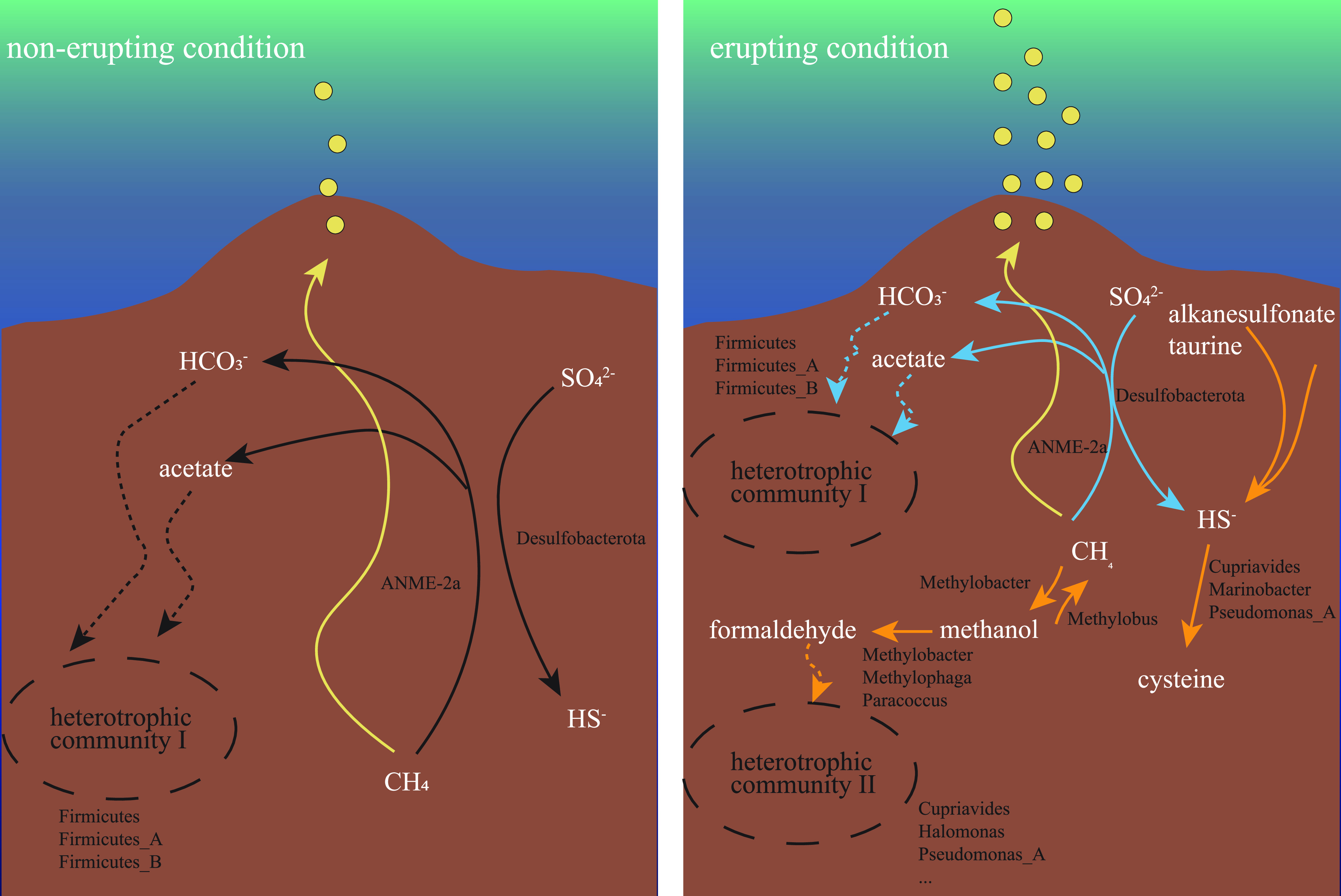
Proposed conceptual model for community shift during the cold-seep eruption. Abundant taxonomy and process before the eruption (left) and during the eruption (right). Color in the right part represents the changes in abundance; blue represents a decrease and orange represents an increase.

A conceptual model was proposed to reflect the community shift at both the taxonomic and metabolic levels (Fig. 5). Under nonerupting conditions, anaerobic oxidation of methane-sulfate reduction (AOM-SR) activity conducted by ANME-2a and SRB was the primary process. AOM-SR was used as the keystone of the system. The products carbon dioxide and acetate would be used by the heterotrophic community, which was composed mainly of *Clostridia* (46.61% to ∼54.13%), followed by *Gammaproteobacteria* (14.54% to ∼21.48%). Under simulated erupting conditions, this relative importance of AOM activity was inhibited, and alternative pathways were observed for both sulfate and methane consumption. In addition to ANME, a portion of methane was oxidized through *Methylobacter* (Cadiz_LYX74) and *Methanolobus*. Methylobacter (Cadiz_LYX74), *Paracoccus* (Cadiz_LYX81) and *Methylophaga* (Cadiz_LYX47), oxidize the product methanol to formaldehyde, which is then assimilated by the emerging heterotrophic community consisting of *Gammaproteobacteria*. Sulfate was transported into cells and assimilatorily reduced to sulfide, alkanesulfonate, and taurine. Above all, a more complex metabolic network was observed.

## DISCUSSION

In this study, by simulating a cold-seep eruption with a continuously running high-pressure bioreactor, the cold-seep microbial community was found to be sensitive to IP and MPP, with the former mainly influencing the biomass. Generally, higher IP would cause major damage to cells and decrease biomass, but this was not the case when it was adjusted to 15 MPa. This result may be caused by the large decrease in ANME-2a, as it was regarded as the sole group to provide energy through the oxidation of methane. The preference of ANME-2a in a certain pressure range has been confirmed by cultivation-based experiments as well as omics-based investigations, and this finding is in accordance with the water depth range of their distribution in nature (32–34). In general, high incubation pressures cause intracellular oxidative stress (35–37). Since this community was incubated under high pressure for years, the dominant groups are piezotolerant and piezophilic and have thus developed strategies to cope with oxidative stress. For example, ANME-2a has genes for cytochrome *c* and membrane-associated coenzyme B-coenzyme M heterodisulfide reductase (HdrDE) in its electron transfer system, and this strain is often found in environments with oxygen intrusion (38, 39). Interestingly, as suggested by our results, the damaging effect of high IP was reversible. After IP was changed to 8 MPa again (L8II), the community seemed to recover from the disturbance. First, the biomass of ANME-2a and SRB increased as much as that of L15 (Fig. S1), and ANME-2a became the dominant archaeal group (Fig. 1b). In addition, positive sulfide production was observed, suggesting that during this incubation, the community was based on AOM-SR activity. Similar taxonomic compositions (Fig. 3) also suggested recovery.

MPP mostly influenced the taxonomic composition and metabolic function (Fig. 3). Under higher MPP, some methane may also be converted to methyl-like substances for emerging heterotrophs to utilize. Though commonly regarded as a reaction for which oxygen is required, this process was demonstrated to occur in anoxic environments (40). Since the high pressure induces the intracellular production of reactive oxygen species, this methyl production process may be more feasible under eruption conditions (35). We consider two sources of methyl-like substances.

### (i) Methane oxidized to methanol by the enzyme PmoABC and further to formaldehyde.

Although regarded as an aerobic reaction, the process from methane to methanol has been observed and reported for several natural anoxic environments and anaerobic incubation conditions (40–46). Most reported methane oxidizers are *Methylobacter*, utilizing nitrate or nitrite to catalyze the conversion from methane to methanol and finally to formaldehyde for utilization. Although there were relatively low concentrations of nitrate and nitrite, this low concentration could lighten the toxic effect of the reduced product, NO. At the same time, *Methylobacter* was reported to perform high-rate methane oxidation in the anoxic hypolimnion stimulated by both sulfate additions (40). An emerging MAG, Cadiz_LYX74, also belongs to the same genus and possesses the same functional gene sets for denitrification (*nirK* and *norB*) and methane oxidation (*pmoABC*), giving the possibility of “aerobic” methane oxidation. The latter also was found to be present and transcribed in the anoxic environment (43, 47, 48). A recent study by He et al. also showed that aerobic gammaproteobacterial methanotrophs dominated in the assimilation of CH_4_, both in their DNA-based stable isotope probing incubations and in the active methane seep (anaerobic region, down to 70 cm deep in sediments) (49). Additionally, the metagenomic result of the heavy DNA showed that *Methylobacter* (27.0%) was the most abundant gammaproteobacterial methanotroph, which was consistent with our results. Moreover, the genes *pmoABC* in the MAG Cadiz_LYX74 were also found to be expressed (with transcripts per kilobase million [TPM] values of 88.99, 94.24, and 1213.12, respectively) in the metatranscriptomic reads collected from the incubation in the same continuous high-pressure bioreactor for 1 year (38).

### (ii) Leakage from activated ANME.

Methanogenesis was observed in three AOM enrichment cultures by Wegener and coworkers. Further enrichments with different substrates suggested that the methane came from methanol and methylamine instead of hydrogen, acetate, and carbon monoxide (50). Those methyl-like substances were proposed as intermediates from methyl-S-CoM (51) in ANME-1, with the absence of the methylenetetrahydromethanopterin reductase (Mer) enzyme from a strict reversal of methanogenesis. A high methane partial pressure would increase the amount of methyl-S-CoM and further stimulate methylated production.

Additionally, 14 out of 16 MAGs that exhibited increasing abundance belonged to the EDD pathway, which could assimilate formaldehyde. Considering the decreasing abundance of genes related to carbon dioxide fixation (Fig. S2) and the decrease in ANME-2a and SRB (Fig. S1), it was proposed that methylated compounds may act as alternative substrates to support the emerging community. Although previous studies showed that a higher MPP would promote AOM activity (19, 52–55), the highest MPP was set to 10 MPa. Shallow marine sediment was incubated at different pressures (pressurized with 100% CH_4_) by Cassarini et al., and there was also a lower sulfide production and AOM rate under higher MPP (20 and 40 MPa) than lower MPP (0.1, 0.45, and 10 MPa), suggesting substrate inhibition during AOM activity (56). The production of methanol also stimulated the emergence of *Methanolobus* (Cadiz_LYX86), whose biomass and abundance increased under eruption conditions (Fig. S1 and Table S3). At the same time, the methanol that was produced could serve as the methanogenic substrate for *Methanolobus*. Methanol was a noncompetitive substrate for methanogens and sulfate reducers (or other heterotrophs), which was observed previously in cold-seep sediments (57, 58). Additionally, a higher methanogenesis rate was observed in the incubation of site GC600 under higher MPP (25 mM methane) than under lower MPP (5 mM methane) (59). The importance of methanol-related methanogenesis has been suggested in both *in situ* and *in vitro* investigations.

### Conclusions.

In conclusion, by enriching the AOM-SR-related microbiome and simulating cold-seep eruption events, we revealed the community drift caused by higher MPP. During geological activities, the community became more diverse, in terms of both taxonomy composition and metabolic potential. AOM-SR activity and the related species were not the exclusive keystones of the cold-seep community, and methane was utilized by multiple pathways, such as oxidation and subsequent formaldehyde assimilation. An unexpected and complex microbial community was revealed under high MPP; in this community, canonic AOM-SR activity was suppressed, and organic carbon production was stimulated. This diversity in taxonomy and metabolic potential would better maintain community stability. In other words, environmental conditions drive the generation of microdiversity (31). These findings emphasize the enormous ecological impact of methane eruptions on cold-seep ecosystems. As a result, more sophisticated *in situ* measurements and modeling of the methane budget in cold seeps that consider AOM-SR suppression by higher MPP and organic carbon derived from incomplete AOM are needed.

## MATERIALS AND METHODS

### Sample site, continuous high-pressure incubation, and activity analysis.

The inoculum was originally obtained from Captain Arutyunov Mud Volcano (35° 39.700′N; 07° 20.012′W) at 1,200 m below the seafloor and had been incubated in a continuous high-pressure bioreactor for 8 years (incubation origin) (19, 60). In this study, six other incubations with different combinations of MPP and IP were carried out (Table 1). Based on the calculated methane affinity for the same ANME-2/SRB community, 8 MPa of methane was provided in the first 4 incubations to achieve the maximum AOM rate (61). The MPP was then adjusted to 12 MPa to mimic a cold-seep eruption. The IP was set to 8 MPa, 15 MPa, and 30 MPa for the simulation of cold seeps at different depths. Every liter of medium consisted of the following: NaCl at 26 g, MgCl_2_·6H_2_O at 5 g, CaCl_2_·2H_2_O at 1.4 g, Na_2_SO_4_ at 1.3 g, NH_4_Cl at 0.3 g, KH_2_PO_4_ at 0.1 g, KCl at 0.5 g, bicarbonate solution at 30 mL, vitamin mixture solution at 1 mL, trace element solution at 1 mL, thiamine solution at 1 mL, and vitamin B_12_ solution at 1 mL. The bicarbonate solution, vitamin solutions, and trace element solution were prepared according to the method of Widdel and Bak (62). The pH of the medium was adjusted to 6.8 by adding sulfuric acid. The medium was prepared under a nitrogen atmosphere; it was first saturated with high-pressure methane and then transferred to the bioreactor at a flow rate of 0.1 mL/min. Incubation was performed at 15°C, and the methane pressure and incubation pressure were changed every 2 months. To monitor AOM-SR activity, the consumption of methane and sulfate, as well as the production of carbon dioxide and sulfide, was analyzed as previously described (63). Of these 4 methods, the quantification of sulfide is the most sensitive for determining AOM activity. All of the sulfide produced comes from sulfate reduction using methane or intermediates derived from methane oxidation as the electron donor. Moreover, there was no sulfide pool in the inlet, so the sulfide production could be precisely quantified. Since we were running a flowthrough system, the sulfide production rate was calculated by measuring the sulfide concentrations from the inlet and outlet of the incubation vessel and considering the incubation volume and flow rate. For acetate measurement, 1 mL of sample filtered through a 0.22-μm membrane filter (Merck Millipore, Billerica, MA) was mixed with 0.25 mL of 50% H_2_SO_4_, and 1 mL of ether solution containing internal standard 2-methylpentanoic acid (50 μg/ml) was then added. Then, the sample was centrifuged at 3,000 rpm for 10 min at 4°C and incubated at 4°C for 30 min. The top layer was injected into a gas chromatograph–triple-quadrupole mass spectrometer (GC-QQQ-MS; Agilent 7890B-7000D; Agilent Technologies, Santa Clara, CA). The detection limit was lower than 80 nM.

### Analysis of 16S rRNA sequencing data.

DNA was extracted and purified according to the modified SDS-based method described by Natarajan et al. (64). Purified DNA was dissolved in 60 μL of double-distilled water (ddH_2_O) and stored at −80°C until use. The V4 region of bacterial 16S rRNA genes was amplified by PCR with the primer pair Bact533F (5′-TGCCAGCAGCCGCGGTAA-3′) and Bact806R (5′-GGACTACCAGGGTATCTAATCCTGTT-3′), while the V4-V5 region of archaeal 16S rRNA genes was amplified with the primer pair Arch516F (5′-TGYCAGCCGCCGCGGTAAHACCVGC-3′) and Arch855R (5′-TCCCCCGCCAATTCCTTTAA-3′) (65). The 50-μL amplification mixture contained 1 μL of each forward and reverse primer, 1 μL of template DNA, 5 μL of 10× *Ex Taq* buffer, 5 μL of 2.5 mM deoxynucleoside triphosphate (dNTP) mix, 0.25 μL of *Ex Taq* polymerase (TaKaRa, Tokyo, Japan) and 39.75 μL of ddH_2_O. The PCR conditions for archaeal 16S rRNA gene amplification were as follows: 94°C for 5 min, followed by 35 cycles at 94°C for 40 s, 60°C for 40 s, and 72°C for 50 s and a final extension at 72°C for 10 min. For bacteria, the PCR conditions were 94°C for 5 min, 25 cycles of 40 s at 94°C, 40 s at 58°C, and 30 s at 72°C, and a final extension for 10 min at 72°C. The PCR products were purified with an E.Z.N.A. gel extraction kit (Omega Bio-Tek, Norcross, GA). The 16S rRNA gene amplicons containing the unique barcodes used for each sample were pooled at equal concentrations and sequenced on an Illumina MiSeq platform using 2 × 250-bp cycles at Shanghai Personal Biotechnology Co., Ltd. (Shanghai, China). The raw sequence reads were quality filtered using an average quality value of 20 during demultiplexing; sequences with a mean quality score of <20, a length <150 bp, or any ambiguities were excluded from the analysis. Raw tags were then generated by merging paired-end (PE) reads with FLASH (version 1.2.7) (66). The resulting sequences were imported to QIIME2 for taxonomic identification. First, the sequences of the corresponding regions from the SILVA 138 database (23) were extracted according to the primers used, and the sequences were then used to train the classifier. Then, chimeric sequences obtained during the PCR process were removed using “qiime vsearch.” The taxonomy of the obtained cluster was determined by “feature classifier” with the classifier. Then, the conflicting sequences for bacteria and archaea were removed.

### Population quantification.

The bacterial and archaeal populations were quantified by quantitative PCR (qPCR). qPCR amplification was performed using a StepOnePlus real-time PCR system (Applied Biosystems, Foster, CA), and all reactions for the bacterial 16S rRNA gene were conducted using SYBR premix *Ex Taq* (TaKaRa, Tokyo, Japan). The primer pair bac331F (5′-TCCTACGGGAGGCAGCAGT-3′) and prokaryotic 797R (5′-GGACTACCAGGGTATCTAATCCTGTT-3′) was used for bacteria, and Uni519F (5′-GCMGCCGCGGTAA-3′) and Arc908R (5′-CCCGCCAATTCCTTTAAGTT-3′) (67) were used for archaea. Each reaction was conducted in triplicate. The qPCR program for bacteria was 50°C for 2 min, 95°C for 2 min, and 40 cycles of 95°C for 15 s and 65°C for 60 s. For archaea, the program was 95°C for 15 min followed by 35 cycles of 95°C for 30 s, 60°C for 30 s, and 72°C for 30 s. The quantification standard consisted of a dilution series (between 1 × 10^3^ and 1 × 10^9^ copies/μL) of a known amount of purified PCR product obtained from sediment environmental DNA. The *R*^2^ value for the standard curve was 0.99, and the amplification efficiency was 95 to 105%. The bacterial cell number was calculated using the bacterial 16S rRNA gene copy number divided by 4, which is the mean 16S rRNA operon number for each incubation calculated by PICRUSt (Phylogenetic Investigation of Communities by Reconstruction of Unobserved States) (68). The cell numbers of ANME-2a, SRB, and *Methanolobus* were calculated using the total archaeal cell number multiplied by the relative abundance in the corresponding community.

### Metagenomic assembly, binning, and annotation.

The 150-bp paired-end raw reads of 7 incubations were first trimmed by BBDuk tools (https://sourceforge.net/projects/bbmap/) with a sequence quality score > 20 and a final minimum length of >90 bp. Then, the reads containing rare kmers (kmer depth ≤ 2) were discarded by tadpole.sh with “tossdepth = 2 ecc=t tossjunk=t mbb = 2.” Next, all reads were merged into SPAdes (69) for coassembly with “--meta --only assembler -k 65,75,96,115,127.” The assembly was filtered for a minimum length of >500 bp using a custom Python script. Then, reads from each incubation were mapped to the filtered assembly separately by BBMap with “k = 13 minid = 0.95 pairlen = 350 rescccuedist = 650.” The mapped file in SAM format was converted to BAM format and sorted by SAMtools (70). The depth of each scaffold in every incubation was determined by the script “jgi_summarize_bam_contig_depths” from MetaBAT2 (71) with the default parameters. Three binning software programs were used to obtain the primary MAG via the depth matrix. For MetaBAT2, different sensitivities (--maxP 60, 75, and 90) and specificities (--minS 60, 75, and 90) were combined. The two marker gene sets (40 and 107) were analyzed by MaxBin (version 2.2.6) (72). CONCOCT analysis (73) was also carried out. Then DAStools (74) was used to integrate the results to calculate an optimized, nonredundant set of MAGs. Quality and taxonomy were determined by CheckM (75) and GTDBtk tools (76) with the GTDB (version r95) (26), respectively. Genes were predicted by Prodigal (77) for the filtered assembly, and those with lengths smaller than 100 bp were discarded. The modified gene set was functionally annotated with an integrated result, with the following priorities: GhostKOALA (78) > emapper (version 2.0.1) against the EggNOG database (version 5) (79, 80) > KofamKOALA (81) (version 1.0.3). FeatureCounts (82) was used to count the read number of each gene, and the TPM value was calculated with a custom Python script.

### Phylogenetic tree construction.

For the tree of MAGs, we randomly chose 1 genome as a reference for each class of the phylum occurring in the MAG taxonomic results in the GTDB. The gene of each reference genome was predicted by Prodigal. Then, fetchMG (83) was used to extract the marker gene for all MAGs and the reference genome. Then, for each marker gene set, alignment and trimming with the same parameter were performed separately by MAFFT-linsi (84) and trimAL (85) with the automated1 model. After that, all alignments were concatenated for each genome. The trimmed alignment was composed of 397 sequences and 8,836 columns. IQTREE (86) was used to construct the phylogenetic tree with the model “LG+I+G4.”

### Community composition and metabolic potential analysis.

Shifts in community composition were analyzed using the ade4 R package based on the read number of archaeal and bacterial genera, the relative abundance of 43 abundant MAGs (relative abundance in any one incubation > 0.5%), and the relative abundance of KEGG orthologs. The result was visualized with the ggplot2 R package.

Statistical analyses of key genes involved in carbon, sulfur, nitrogen, and ATP metabolism between incubations L15 and H15 were performed by pairwise comparisons of their abundances using two-sided Fisher’s exact test with confidence intervals at 95% significance and the Newcombe-Wilson method and Benjamini-Hochberg false-discovery rate (FDR) multiple-test correction in STAMP (87). Only the module with corrected *q* value of <0.05 was shown.

For each MAG, the metabolic potential was evaluated by the completeness of every module, which was determined by the presence/absence of KO terms with a custom Python script. The score was modified by the following criterion: (0.75, 1) was adjusted to 1, (0.2, 0.75) was adjusted to 0.5, and (0, 0.2) was adjusted to 0.

### Metatranscriptome sequencing and mapping.

All metatranscriptomic reads were first filtered by BBDuk tools and then aligned to a combined rRNA database from SILVA and Rfam (88) using Bowtie2 (89). The unaligned mRNA reads were mapped to the assembled scaffolds. The expression of each gene was calculated as described above.

### Data availability.

The 16S rRNA gene amplicon sequencing data and metagenomic sequencing data have been deposited in the NODE database (https://www.biosino.org/node/) under project OEP001382. All metagenome-assembled genomes have been deposited in eLMSG (eLibrary of Microbial Systematics and Genomics [https://www.biosino.org/elmsg/index]) under accession numbers LMSG_G000000632.1 to LMSG_G000000741.1.
