# Supplementary figures and images for: Stimulated Organic Carbon Cycling and Microbial Community Shift Driven by a Simulated Cold-Seep Eruption
Source: mBio. 2022 Mar 1;13(2):e00087-22. doi: 10.1128/mbio.00087-22 (PMC8941925; doi:10.1128/mbio.00087-22)

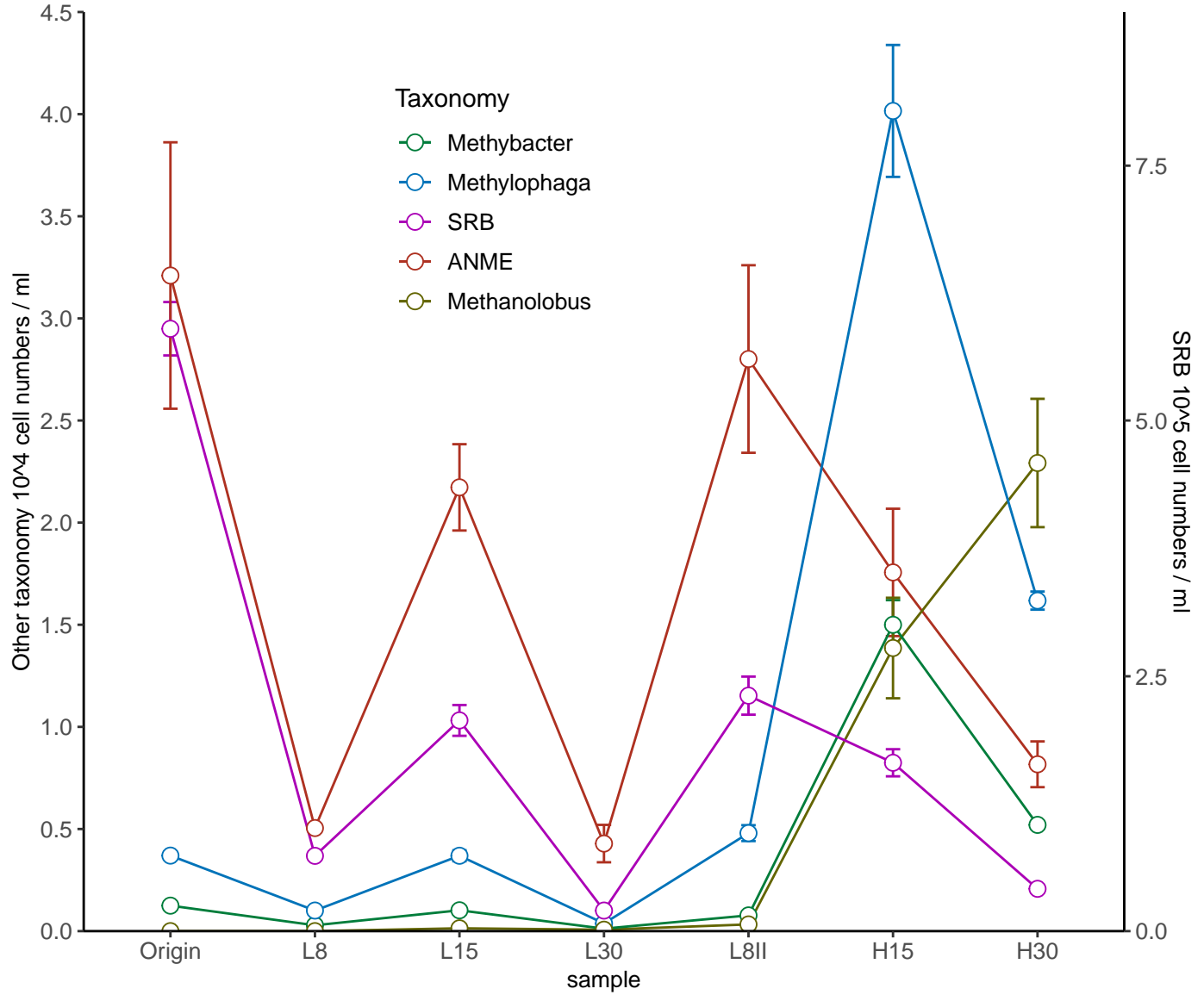

Supplement: FIG S1 [file mbio.00087-22-sf001.pdf]

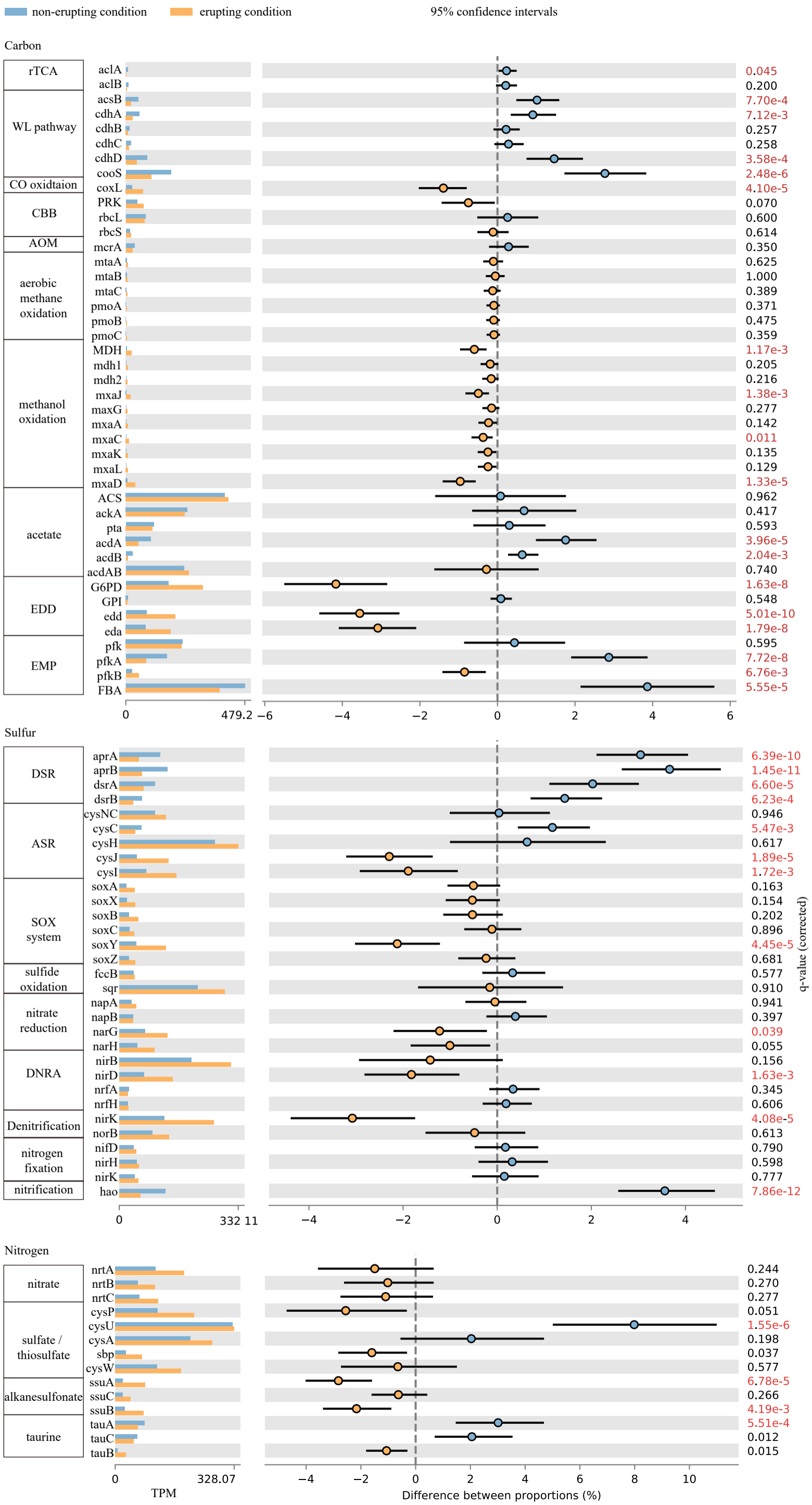

Supplement: FIG S2 [file mbio.00087-22-sf002.pdf]
